# “Popping the Ion‐Basket”: Enhancing Thermoelectric Performance of Conjugated Polymers by Blending with Latently Dissociable Perovskite Quantum Dots

**DOI:** 10.1002/advs.202412663

**Published:** 2025-01-22

**Authors:** Hansol Lee, Hoimin Kim, Haedam Jin, Seungju Kang, Tae Woong Yoon, Dongki Lee, Guobing Zhang, Min Kim, Boseok Kang

**Affiliations:** ^1^ Department of Chemical and Biological Engineering Gachon University Seongnam 13120 Republic of Korea; ^2^ SKKU Advanced Institute of Nanotechnology (SAINT) and Department of Nano Science and Technology Sungkyunkwan University Suwon 16419 Republic of Korea; ^3^ Graduate School of Integrated Energy‐AI Jeonbuk National University Jeonju 54896 Republic of Korea; ^4^ Department of Nanotechnology and Advanced Materials Engineering Sejong University 209 Neungdong‐ro, Gwangjin‐gu Seoul 05006 Republic of Korea; ^5^ National Engineering Lab of Special Technology Academy of Optoelectronic Technology Anhui Province Key Laboratory of Measuring Theory and Precision Instrument School of Chemistry and Chemical Engineering Key Laboratory of Advance Functional Materials and Devices of Anhui Province Hefei University of Technology Hefei 230009 China; ^6^ School of Chemical Engineering, Research Institute for Materials and Energy Sciences Jeonbuk National University Jeonju 54896 Republic of Korea; ^7^ Department of JBNU‐KIST, Industry‐Academia Convergence Research Jeonbuk National University Jeonju 54896 Republic of Korea; ^8^ Department of NanoEngineering and Department of Semiconductor Convergence Engineering Sungkyunkwan University Suwon 16419 Republic of Korea

**Keywords:** conjugated polymers, doping efficiency, organic thermoelectrics, perovskite quantum dots, Seebeck coefficient

## Abstract

A novel additive method to boost the Seebeck coefficient of doped conjugated polymers without a significant loss in electrical conductivity is demonstrated. Perovskite (CsPbBr_3_) quantum dots (QDs) passivated by ligands with long alkyl chains are mixed with a conjugated polymer in a solution phase to form polymer‐QD blend films. Solution sequential doping of the blend film with AuCl_3_ solution not only doped the conjugated polymer but also decomposed the QDs, resulting in a doped conjugated polymer film embedded with separated ions dissociated from the QDs. For the doped polymer‐molten QD blend films with the optimal QD content, it is found that a greatly enhanced Seebeck coefficient is achieved compared to that of the doped polymer film without QDs, while the doping level and electrical conductivity are not significantly reduced by the QD incorporation. Consequently, the power factor is enhanced, reaching a remarkably high value of up to 401.9 µW m^−1^ K^−2^ (≈155% increase with the QDs). The applicability of this method to a variety of conjugated polymers is also demonstrated. The enhancement in the Seebeck coefficient is attributed to ion‐induced local variations in the polymer work function, which generates an internal energy barrier for charge transport and causes an energy filtering effect.

## Graphical abstract

A novel strategy to enhance the thermoelectric performance of conjugated polymers through the incorporation of latently dissociable perovskite quantum dots is demonstrated. The quantum dots dissociate during the sequential solution doping of the conjugated polymers, resulting in a significant increase in the Seebeck coefficient of the polymer compared to the case without quantum dots.

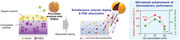

## 1 Introduction

Thermoelectric materials produce electricity from a temperature difference, enabling the environment‐friendly conversion of waste heat into electrical energy. Thermoelectric materials based on conjugated polymers have been intensively investigated because of their advantages in flexible devices and solution‐based, low‐cost processes.^[^ ^1^, ^2^ ^]^ However, despite tremendous research efforts, their thermoelectric performance is still not satisfactory for commercialization, necessitating further development of high‐performance materials and processing methods.^[^ ^2^, ^3^, ^4^, ^5^ ^]^

The thermoelectric performance of a material is generally characterized by its thermoelectric figure of merit *zT*, which is a dimensionless parameter. *zT* is defined by *zT* = *S* ^2^ *σT*/*κ*, where *S* is the Seebeck coefficient, *σ* is the electrical conductivity, *κ* is the thermal conductivity of the material, and *T* is the temperature. Usually, the factor *S* ^2^ *σ*, called thermoelectric power factor (PF), is simply used to present the thermoelectric performance of materials. Studies on thermoelectric conjugated polymers have mainly focused on strategies to improve their electrical conductivity because their PFs are usually limited by their low electrical conductivity.^[^ ^5^, ^6^, ^7^, ^8^ ^]^ Electrical conductivity of conjugated polymers can be increased by chemical doping, which increases the concentration of charge carriers within the polymer via electron transfer between the polymer chain and dopant.^[^ ^9^, ^10^, ^11^ ^]^ However, increasing the charge carrier concentration induces a simultaneous decrease in the Seebeck coefficient of the polymer; thus, maximizing the thermoelectric performance only by doping is always limited by the trade‐off between the electrical conductivity and Seebeck coefficient.^[^ ^10^, ^12^, ^13^, ^14^ ^]^

Recently, several blending strategies have been reported to improve the thermoelectric performance of conjugated polymers.^[^ ^3^, ^15^, ^16^ ^]^ Blending conjugated polymers with heterogeneous materials such as other conjugated molecules and inorganic nanomaterials may provide further room for tuning the thermoelectric properties of the blends. In particular, this strategy often leads to a decoupled increase in the Seebeck coefficient and electrical conductivity, overcoming the tradeoff relationship between them. For example, density of states (DOS) engineering demonstrated by blending another conjugated polymer having a different highest occupied molecular orbital (HOMO) energy level with a host conjugated polymer can effectively increase the Seebeck coefficient of the blend by maximizing the separation of the transport level (*E*_tr_) and Fermi level (*E*_F_) by locating them at different HOMO DOS of the respective polymers.^[^ ^16^, ^17^, ^18^, ^19^, ^20^ ^]^ Addition of conjugated small molecules was also shown to enhance the Seebeck coefficient of a host conjugated polymer, by splitting the polaron level of the conjugated polymer via π–π overlapping of the small molecule and conjugated polymer and thus increasing the separation between *E*_tr_ and *E*_F_.^[^ ^21^ ^]^ The addition of inorganic nanomaterials such as carbon nanotubes,^[^ ^3^ ^]^ Bi_2_Te_3_,^[^ ^22^ ^]^ Te nanowires,^[^ ^23^ ^]^ carbon dots,^[^ ^24^ ^]^ and MXenes^[^ ^25^, ^26^ ^]^ can increase the Seebeck coefficient via the energy‐filtering effect,^[^ ^27^ ^]^ which modulates *E*_tr_ by excluding low‐energy charge carriers from electrical conduction via internal potential barriers within the blend.^[^ ^24^, ^28^ ^]^

Although various materials have been used for the blending strategy, ionic compounds are rarely used because of their limited processability in common non‐polar organic solvents. The low solubility of ionic compounds in organic solvents necessitates an increase in the temperature of the blend solution, employing co‐solvent systems, or limiting the amount of added ionic compounds to a small quantity to ensure processability.^[^ ^29^, ^30^ ^]^ Consequently, studies investigating the addition of ionic compounds to improve the performance of thermoelectric conjugated polymers are scarce for polymers other than poly(3,4‐ethylenedioxythiophene):polystyrene sulfonate (PEDOT:PSS); even in studies based on PEDOT:PSS, the focus tends to be on the Soret effect of mobile ions rather than the electronic thermoelectric effect of the conjugated polymer itself.^[^ ^31^, ^32^, ^33^, ^34^ ^]^ However, as demonstrated in a recent study,^[^ ^30^ ^]^ the incorporation of ionic compounds can also be an effective approach to boost the electronic thermoelectric performance of conjugated polymers. Thus, further studies on the development of proper materials and processing methods for this approach are required for the development of highly efficient thermoelectric polymer composites.

In this study, we demonstrated a novel method for utilizing ionic compounds to improve the thermoelectric performance of conjugated polymer films. A halide perovskite (CsPbBr_3_) quantum dot (QD) was used as a model ionic compound in this study. The passivation of CsPbBr_3_ QDs with ligands having long alkyl chains enabled the incorporation of QDs into conjugated polymer thin films by simply blending the QDs and conjugated polymers in common non‐polar organic solvents. Sequential solution doping of the blend film with AuCl_3_ led to the decomposition of the QDs, resulting in the embedding of ions dissociated from the molten QDs into the polymer film. The presence of ions did not significantly affect the doping level and led to a mild decrease in the electrical conductivity of the doped polymer film. However, it greatly enhanced the Seebeck coefficient of the doped polymer film, contributing to a higher thermoelectric PF of the polymer compared to that of the doped polymer film without QD. This method worked successfully on several conjugated polymers, suggesting its general applicability to various conjugated polymers. The Seebeck coefficient enhancement was explained by the ion‐induced local variations in the work function of the doped polymer, which generated an internal energy barrier for charge transport within the doped polymer film and caused an energy filtering effect that boosted the Seebeck coefficient.

## 2 Results and Discussion

### 2.1 Synthesis of Perovskite QDs and Its Incorporation into Conjugated Polymer Thin Films

**Figure 1:**
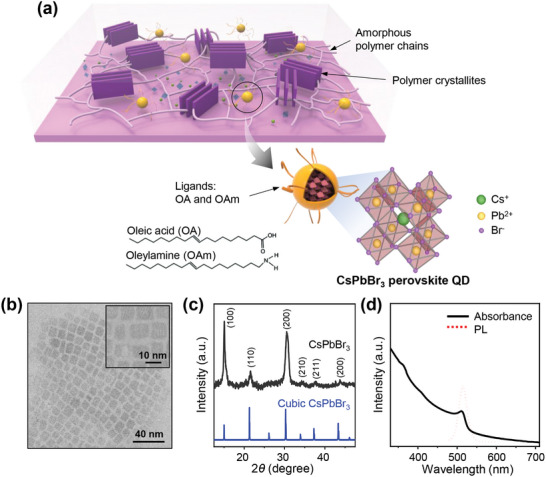
Schematic of the overall experimental system and characterization of CsPbBr_3_ QD used in this study. a) Schematic drawing of polymer‐QD composite films and the detailed structure of CsPbBr_3_ QD. The surface of CsPbBr_3_ QD is passivated by ligands with long alkyl chains (OA and OAm) to ensure solubility in common organic solvents. b) TEM images of the synthesized CsPbBr_3_ QDs, which have a cubic shape with an average side length of ≈10 nm. c) X‐ray diffraction pattern of the synthesized CsPbBr_3_ QD (black). The pattern of cubic CsPbBr_3_ is also shown for reference (blue). d) UV–vis absorption spectra (black solid line) and PL spectra (red dotted line) of the film of CsPbBr_3_ QD.

**Figure** **1**a shows the schematic of the overall experimental system used in this study. QDs (yellow spheres) are dispersed and embedded within a conjugated polymer thin film (purple matrix), as an ionic additive that can enhance the thermoelectric performance of the conjugated polymer. The QDs are surrounded by ligand molecules with long alkyl chains, so they can be solubilized in common non‐polar organic solvents and can be easily incorporated within the polymer film. When the polymer‐QD composite film is sequentially doped with a dopant solution, the QDs are dissociated into separate ionic species, resulting in the enhancement of the thermoelectric performance of the polymer‐QD composite film.

An all‐inorganic perovskite cesium lead halide QD, cesium lead bromide (CsPbBr_3_) QD, was used as a model additive for this study (Figure 1a). High‐quality CsPbBr_3_ quantum dots were synthesized using a previously reported method.^[^ ^35^ ^]^ Cesium stearate was hot‐injected into PbBr_2_ solution at 170 °C to form CsPbBr_3_ QDs. The surfaces of the synthesized QD were adsorbed by ligands such as oleylamine and oleic acid (OAm and OA, respectively), which enabled the stable dispersion of the QDs in various non‐polar organic solvents such as chloroform, chlorobenzene, and toluene. Further details on the preparation of the CsPbBr_3_ QD are reported in the Experimental Section of the Supporting Information. The synthesized CsPbBr_3_ QD had a cubic shape with a characteristic length of ≈10 nm (Figure 1b). The X‐ray diffraction result of the QDs matched well with those of previous reports (Figure 1c), indicating its cubic crystal structure with a lattice parameter of 5.8 Å.^[^ ^36^, ^37^, ^38^ ^]^ The UV–vis absorption and photoluminescence (PL) spectra of the CsPbBr_3_ QD were characterized (Figure 1d). Neat CsPbBr_3_ QD films were obtained by spin coating a solution in which the QDs were dispersed in toluene. The absorption spectrum of the as‐cast CsPbBr_3_ QD film shows an absorption edge at 530 nm, which indicates an optical bandgap of 2.34 eV. The PL spectrum (excited at 365 nm) of the film exhibited a peak at 514 nm. These results agree well with those of previous reports on CsPbBr_3_ QDs with sizes of ≈10 nm.^[^ ^35^, ^38^, ^39^ ^]^

**Figure 2:**
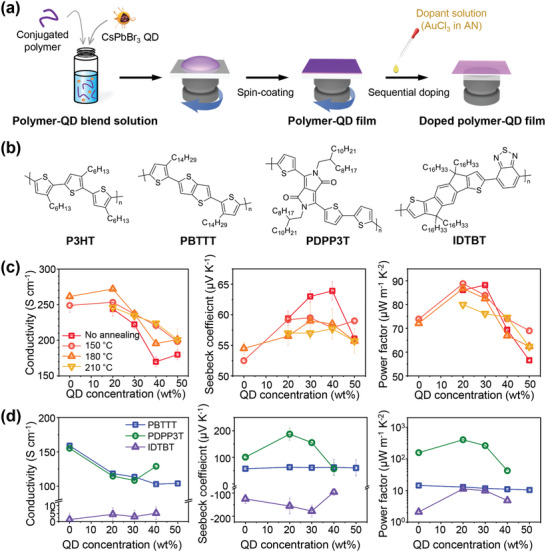
Preparation of polymer‐QD blend films and characterization of their thermoelectric performance after doping. a) Schematics of experimental procedure. Conjugated polymer and perovskite QD were blended and spin‐coated to obtain a polymer‐QD blend film. The blend film was then sequentially doped with AuCl_3_ solution. b) Molecular structure of conjugated polymers used in this study. c) Thermoelectric properties of doped P3HT‐QD film as a function of the QD content within the blend. The P3HT‐QD film was thermally annealed at different temperatures before sequential doping to optimize its thermoelectric performance. d) Thermoelectric properties of other doped polymer‐QD films as a function of the QD content.

To incorporate CsPbBr_3_ QDs into conjugated polymer thin films, the conjugated polymers, and CsPbBr_3_ QD were blended in chlorobenzene and spin‐coated onto substrates (**Figure** **2**a). The long alkyl chains of the OAm and OA ligands attached to the QD surface provided compatibility between the QDs, the solvent, and the conjugated polymer. Four different conjugated polymers (Figure 2b), poly(3‐hexylthiophene) (P3HT), poly[2,5‐bis(3‐tetradecylthiophen‐2‐yl)thieno[3,2‐b]thiophene] (PBTTT), poly{2,2′‐[(2,5‐bis(2‐octyldodecyl)‐3,6‐dioxo‐2,3,5,6‐ tetrahydropyrrolo[3,4‐c]pyrrole‐1,4‐diyl)dithiophene]‐5,5′‐diyl‐alt‐thiophen‐2,5‐diyl} (PDPP3T), and 2,1,3‐benzothiadiazole‐4,7‐diyl‐co‐4,4,9,9‐tetrahexadecyl‐4,9‐dihydro‐s‐indaceno[1,2‐b:5,6‐b‘]dithiophene‐2,7‐diyl (IDTBT) were used as the host conjugated polymer, to test the general applicability of the QD‐blending strategy in improving thermoelectric performance of various doped conjugated polymers with different molecular structures. Among these polymers, P3HT was used primarily for the analyses performed in this study, which aimed to understand how the incorporation of QD affects the thermoelectric properties of conjugated polymers.

### 2.2 Effects of Perovskite QD Incorporation on Thermoelectric Properties of Doped Conjugated Polymers

The CsPbBr_3_ QD‐containing polymer thin films were doped using the solution‐sequential doping method (Figure 2a). AuCl_3_ was used as the dopant, and acetonitrile (AN) was used as the solvent for the dopant solution. Before doping, the polymer films were thermally annealed at various temperatures to optimize their thermoelectric properties. The dopant solution was then dripped onto the polymer film and held there for 1 min to allow the dopants to penetrate the entire film. After spinning to remove the remaining dopant solution on the film, the doped polymer film was thermally annealed at a mild temperature (80 °C) for complete drying of the film. Experimental details on the preparation of the blend films and the doping process are provided in the Supporting Information.

The thermoelectric properties of doped conjugated polymer films were investigated. The undoped polymer films did not show measurable electrical conductivities or Seebeck coefficients even though they contained CsPbBr_3_ QDs. The electrical conductivities and Seebeck coefficients of the doped polymer films were measured as functions of the CsPbBr_3_ QD content, and the corresponding PFs were calculated (Figure 2c,d). In the case of doped P3HT (Figure 2c, **Table** **1**, and Table S1, Supporting Information), the electrical conductivity increased slightly with the incorporation of 20 wt% QD, and a further increase in the QD content resulted in a reduction in the electrical conductivity. This trend was observed for all P3HT films thermally annealed at different temperatures. The Seebeck coefficient gradually increased with increasing QD content, reaching a peak at a specific QD content between 20 and 40 wt%, depending on the thermal annealing temperature of the P3HT film, after which the Seebeck coefficient decreased. Consequently, PF showed maximum values at 20–30 wt% QD content for P3HT films annealed at different temperatures. The highest PF of 88.8 µW m^−1^ K^−2^ was obtained for the 150 °C‐annealed P3HT film containing 20 wt% CsPbBr_3_ QD. This value represents a 20% improvement compared to the highest PF value of doped P3HT without QD (i.e., 73.9 µW m^−1^ K^−2^). The improvement in the PF was mainly driven by the increase in the Seebeck coefficient owing to the QD incorporation, as indicated by the dependence of the electrical conductivity and Seebeck coefficient on the QD content. Note that when only the ligand OA is added to the P3HT, the OA molecules did not improve the conductivity nor the Seebeck coefficient of P3HT film (Table S2, Supporting Information), indicating that the QD‐induced improvement in the thermoelectric performance of doped P3HT does not result from the ligand molecules.

**Table 1:** Thermoelectric properties of AuCl_3_‐doped polymer‐QD films with different QD contents.

Polymer	QD content [wt%]	Electrical Conductivity [S cm^−1^]	Seebeck coefficient [µV K^−1^]	Power factor [µW m^−1^ K^−2^]
P3HT	0	246.0 ± 10.2	52.5 ± 0.3	73.9
	20	253.4 ± 2.5	59.2 ± 1.6	88.8
	30	236.9 ± 1.4	59.5 ± 0.3	83.9
	40	222.8 ± 6.3	57.9 ± 0.2	74.7
	50	198.2 ± 2.6	59.0 ± 0.2	69.0
PBTTT	0	159.0 ± 3.2	57.2 ± 0.1	14.4
	20	118.4 ± 4.4	62.8 ± 0.1	12.9
	30	113.4 ± 4.9	61.1 ± 1.1	11.7
	40	103 ± 3.5	62.1 ± 0.3	10.9
	50	104 ± 3.2	60.1 ± 0.3	10.4
PDPP3T	0	155.2 ± 3.2	100.7 ± 0.5	157.3
	20	114.6 ± 2.3	187.3 ± 2.3	401.9
	30	108.4 ± 1.5	155.4 ± 1.0	262.0
	40	129.0 ± 2.7	57.2 ± 2.3	42.2
IDTBT	0	1.33 ± 4.0	−125.3 ± 2.0	2.1
	20	4.6 ± 4.2	−156.1 ± 3.4	11.2
	30	3.1 ± 3.9	−177.9 ± 2.6	9.8
	40	5.3 ± 4.3	−95.49 ± 1.6	4.8

Similar improvements in the thermoelectric performance were observed for other conjugated polymers (Figure 2d and Table 1). In the case of doped PBTTT and PDPP3T, the electrical conductivity decreased with increasing CsPbBr_3_ QD content, as in the case of doped P3HT. In contrast, the electrical conductivity of IDTBT increases slightly with increasing QD content. The dependence of the Seebeck coefficient of the doped polymer films on the QD content is similar to that of P3HT. That is, the magnitudes of the Seebeck coefficients of PBTTT, PDPP3T, and IDTBT increased as the QD content increased, peaked at 20–30 wt% QD content, and then decreased again with further increases in the QD content. The extent of the increase in the Seebeck coefficient varied among the different polymers. Especially, PDPP3T exhibited a dramatic increase, reaching a maximum Seebeck coefficient of 187.3 µV K^−1^, which was ≈86% higher than the value without QD (100.7 µV K^−1^). In contrast, for PBTTT, it showed a maximum value of 62.8 µV K^−1^, which was only ≈10% higher than the value without QD (57.2 µV K^−1^). In the case of IDTBT, the Seebeck coefficient was negative, and the incorporation of QD resulted in more negative values. The Seebeck coefficient of the doped IDTBT was −125.3 µV K^−1^ without the QD, and it decreased to −177.9 µV K^−1^ when 30 wt% QD was included in the film. Owing to the changes in electrical conductivity and Seebeck coefficient upon QD incorporation, the thermoelectric performances of PDPP3T and IDTBT improved significantly. The maximum PF of PDPP3T was 157.3 µW m^−1^ K^−2^ without QD but was increased to 401.9 µW m^−1^ K^−2^ (an increase of 155%) by the incorporation of 20 wt% QD. The maximum PF of IDTBT was 2.1 µW m^−1^ K^−2^ without QD but was increased to 11.2 µW m^−1^ K^−2^ by the incorporation of 20 wt% QD. PBTTT showed a slight decrease in PF with increasing QD content because the considerable decrease in electrical conductivity compensated for the mild increase in the Seebeck coefficient. To the best of our knowledge, the PF of 401.9 µW m^−1^ K^−2^ of the PDPP3T we achieved in this study is one of the highest values among previously reported conjugated polymers, excluding those based on PEDOT:PSS. For reference, the highest PF of PDPP3T among previous reports was 247 µW m^−1^ K^−2^, which was reported by I. H. Jung et al. upon FeCl_3_ doping.^[^ ^40^ ^]^ Our results show that the incorporation of CsPbBr_3_ QDs inside conjugated polymer films via a simple blending process provides further opportunities to enhance their thermoelectric performance upon doping and that this strategy can be generally applied to a variety of conjugated polymers with different molecular structures.

### 2.3 Decomposition of Perovskite QDs by Sequential Doping of Polymer‐QD Blend Films

**Figure 3:**
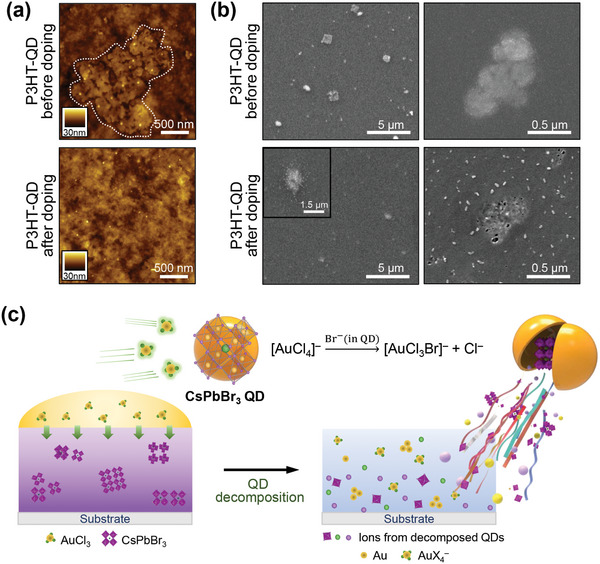
Decomposition of CsPbBr_3_ QDs during sequential doping process. a) AFM height images of P3HT‐QD blend film before and after sequential doping with AuCl_3_ solution. In the image of the film before doping, a white dotted line indicates a QD aggregate embedded in the P3HT matrix. b) SEM images of the P3HT‐QD blend film before and after sequential doping. c) Schematic illustration of the decomposition process of the QD by AuCl_3_ solution.

To unravel how CsPbBr_3_ QDs affect the thermoelectric properties of the host‐conjugated polymers after the sequential doping process, detailed structural analyses of the polymer‐QD films were performed. Atomic force microscopy (AFM) was performed to obtain a height image of the P3HT‐QD blend film before and after sequential doping (**Figure** **3**a). The P3HT‐QD film before sequential doping showed aggregates of CsPbBr_3_ QDs embedded in the P3HT matrix (Figure 3a, top). After the film was in contact with the dopant solution during sequential doping, the QD aggregates disappeared and could not be observed in the AFM height images (Figure 3a, bottom), indicating that the QDs were decomposed by the dopant solution. The decomposition of the QDs by the dopant solution was confirmed by elemental distribution analysis using scanning electron microscopy (SEM) and energy‐dispersive X‐ray spectroscopy (EDS) (Figure 3b; Figure S1, Supporting Information). Here, the doping concentration and time were controlled to capture images of the CsPbBr_3_ QD undergoing decomposition before complete decomposition. In the secondary electron image of the samples, QD aggregates were observed in the undoped P3HT‐QD film (Figure 3b, top), whereas QD aggregates gradually diminished in size and became indistinct in shape in the doped P3HT‐QD film (Figure 3b, bottom), indicating the decomposition of QDs by the AuCl_3_ solution. Elemental maps of the P3HT‐QD films confirmed the presence of CsPbBr_3_ QDs within the films (Figure S1, Supporting Information); the locations of the intense EDS signals of Pb, Cs, Br, and Cl ions corresponded well with the regions where the aggregates were observed in the secondary electron images. Note that the EDS signals from Cs and Br in the doped P3HT‐QD film were much weaker than those of Pb and Cl and were not detectable in the elemental maps. Interestingly, although the CsPbBr_3_ QD themselves did not contain Cl, the elemental map of the QD undergoing decomposition showed an intense Cl signal around the QD. We speculate that this arises from the halide exchange reaction between AuCl_3_ and the CsPbBr_3_ QD, which will be discussed later in this section.

The decomposition of the CsPbBr_3_ QDs by the AuCl_3_ dopant solution was further confirmed by changes in the optical properties of the neat CsPbBr_3_ QD films before and after treatment with the AuCl_3_ solution (Figure S2, Supporting Information). When a neat CsPbBr_3_ QD film was treated with the AuCl_3_ dopant solution in the same manner as the sequential doping of the polymer‐QD films, the UV–vis absorption and PL spectra were almost completely attenuated, indicating the destruction of the structure of the CsPbBr_3_ QDs. The disappearance of the absorption and emission peaks is not simply due to the QDs being washed away from the substrate. Instead, the QDs decomposed and transformed into other chemical species through chemical interactions between the QDs and dopant solution, as supported by the SEM images and X‐ray diffraction results (Figures S3, S4, Supporting Information, respectively). The QD film after the AuCl_3_ solution treatment clearly showed the presence of residual chemical products (Figure S3, Supporting Information). The 2D grazing‐incidence X‐ray diffraction (2D GIXD) patterns of the residual products were completely different from those of the neat CsPbBr_3_ QD film (Figure S4, Supporting Information), confirming that the resulting products were different from the CsPbBr_3_ QD, although their exact identification was challenging.

Interestingly, the CsPbBr_3_ QDs did not decompose when pure AN (the solvent in the dopant solution). The 2D GIXD pattern of the pure AN‐treated neat CsPbBr_3_ QD film was nearly identical to that of the untreated film (Figure S4, Supporting Information), indicating that the QDs were not decomposed by pure AN. This implies that the decomposition of the CsPbBr_3_ QDs requires not only the solvent of the dopant solution but also the AuCl_3_ dopant. AN is widely used as an antisolvent for CsPbBr_3_ QD because of its low solubility for CsPbBr_3_ QDs.^[^ ^41^, ^42^ ^]^ We attributed the decomposition of the CsPbBr_3_ QDs in the AN‐based AuCl_3_ solution to the chemical interaction between the QD bromide ions and AuCl_3_ molecules. AuCl_3_ is a Lewis acid with a d^8^ soft metal (Au(III)) center that tends to form square‐planar complexes with halide ions. The complexation strength of Au(III) with Br^−^ ion is greater than that with Cl^−^,^[^ ^43^ ^]^ so the chloride ligands in AuCl_4_^−^ ions formed by the doping process will be replaced by Br^−^ ions of the CsPbBr_3_ QDs, destroying the crystalline structure of CsPbBr_3_ QDs. This decomposition mechanism explains the intense EDS signals of Cl atoms observed in Figure S1 (Supporting Information). We schematically summarize the decomposition process of CsPbBr_3_ QDs in polymer‐QD films during the sequential doping process (Figure 3c).

**Figure 4:**
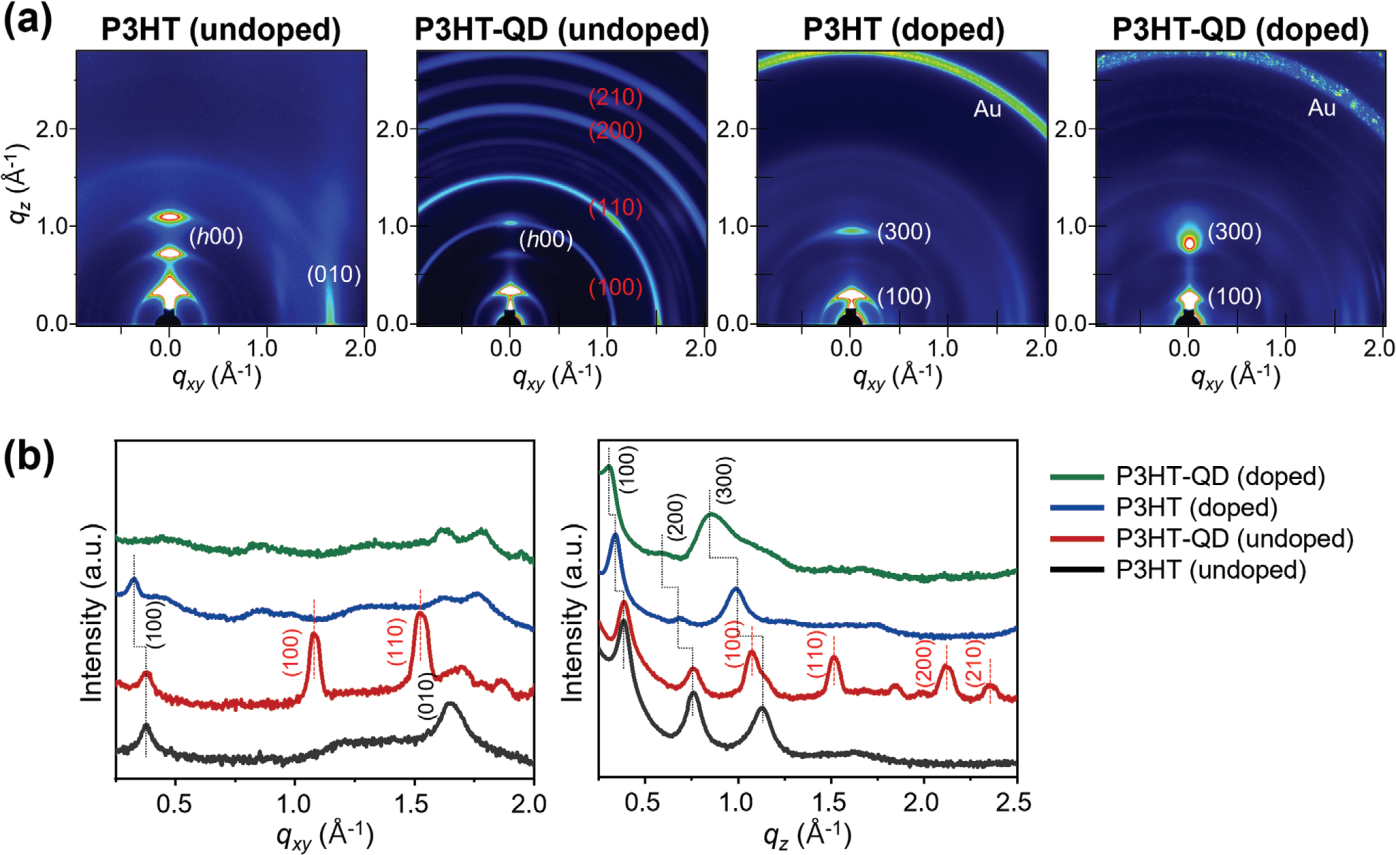
Effect of incorporation of CsPbBr_3_ QD in P3HT film microstructure. a) 2D‐GIXD images of P3HT and P3HT‐QD films before and after sequential doping with AuCl_3_ solution. b) The corresponding 1D line profiles along the in‐plane (left) and out‐of‐plane (right) directions.

Finally, we investigated how the incorporation of CsPbBr_3_ QDs affects the crystalline structure of the undoped and doped P3HT films (**Figure** **4** and **Table** **2**). In the undoped P3HT‐QD film, the presence of QDs slightly attenuated the diffraction intensities of the P3HT (*h*00) and (010) peaks but did not affect the peak positions. This indicates that the incorporation of QDs lowers the crystallinity of P3HT but does not affect the packing structure of the polymer chains. Diffraction peaks from the QDs were observed in the ring patterns, indicating the random isotropic orientational distribution of the QDs within the P3HT film. In doped P3HT film without QD, the (*h*00) and (010) diffraction intensities were reduced, especially for the (200) and (010) peaks, compared to the undoped P3HT film, with the simultaneous appearance of a new weak peak positioned at *q*_xy_ = ≈0.84 Å^−1^ in the in‐plane direction. Furthermore, the lamellar spacing increased from 16.4 Å for undoped P3HT to 18.6 Å for doped P3HT, and the π–π stacking distance decreased from 3.93 Å for undoped P3HT to 3.82 Å for doped P3HT. All of these changes are in good agreement with a previous report and are attributed to the generation of AuCl_4_^−^ ions and Au nanoparticles by AuCl_3_ doping, which increased the lamellar spacing of the P3HT crystallites by positioning them in the hexyl side chain regions.^[^ ^44^ ^]^ In the case of doped P3HT‐QD film, the diffraction pattern resembled that of doped P3HT film without QD, but the lamellar spacing was further increased to 20.9 Å and the peak at *q*_z_ = ≈0.85 Å^−1^ in the out‐of‐plane direction was much broadened. The broadening of the peak at *q*_z_ = ≈0.85 Å^−1^ is because the peak is the superposition of the (300) peak of doped P3HT and the peak of a decomposition product of the QD. Note that a broad peak centered at *q*_z_ = ≈0.85 Å^−1^ has been already seen in the GIXD pattern of AuCl_3_ solution‐treated CsPbBr_3_ QD film (Figure S4, Supporting Information). The presence of this peak in the doped P3HT‐QD film implies the existence of the same ionic products within the film but with a much smaller size or content, as indicated by the much weaker diffraction peak intensity. Importantly, the increased lamellar spacing of the doped P3HT‐QD compared to that of the doped P3HT without QD suggests the incorporation of a greater number of chemical species, that is, dopant anions and ions generated from the decomposition of CsPbBr_3_ QDs, in the side chain regions of P3HT.

**Table 2:** Detailed information on the crystalline structure of P3HT and P3HT‐QD films.

Crystallographic parameters		P3HT		P3HT‐QD	
		Undoped	Doped	Undoped	Doped
P3HT Lamellar stacking (100)	*q* [Å^−1^]	0.383	0.338	0.387	0.301
	*d*‐spacing [Å]	16.4	18.6	16.2	20.87
	FWHM [Å^−1^]	0.053	0.039	0.042	0.062
	Coherence length [Å]	106	145	134.4	91.5
P3HT π–π stacking (010)	*q* [Å^−1^]	1.60	1.65	1.67	1.67
	*d*‐spacing [Å]	3.93	3.82	3.77	3.77
	FWHM [Å^−1^]	0.402	0.328	0.384	0.328
	Coherence length [Å]	17.1	21.4	18.3	21.4
CsPbBr_3_ QD (100)	*q* [Å^−1^]	1.07	‐	‐	‐
CsPbBr_3_ QD (110)	*q* [Å^−1^]	1.52	‐	‐	‐
CsPbBr_3_ QD (200)	*q* [Å^−1^]	2.12	‐	‐	‐
CsPbBr_3_ QD (210)	*q* [Å^−1^]	2.35	‐	‐	‐

### 2.4 Origin of Perovskite QD‐Induced Enhancement of Thermoelectric Performance

As shown in Section 2.2, the incorporation of an optimal amount of CsPbBr_3_ QDs decreased the electrical conductivity and increased the Seebeck coefficient of the doped polymer films. The decrease in the electrical conductivity was relatively small, whereas the increase in the Seebeck coefficient was significant, resulting in an overall increase in PF. We note that the Seebeck coefficient enhancement is irrelevant to thermal diffusion (i.e., the Soret effect) of ions from decomposed QDs because in our experimental system, the ions from decomposed CsPbBr_3_ QDs are embedded in a solid‐state polymer film, which would not provide sufficient ion mobility to generate Soret effect.^[^ ^33^, ^34^, ^45^, ^46^ ^]^

**Figure 5:**
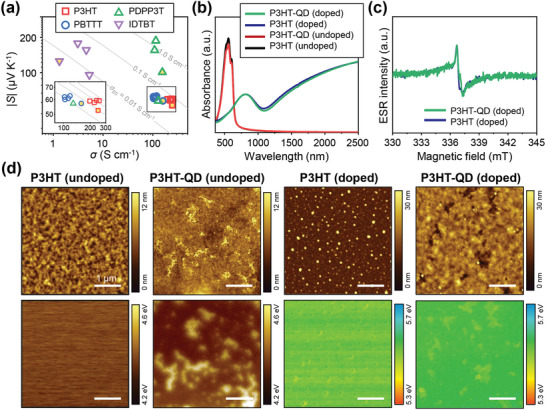
Characterizations on doped polymer films with and without QD. a) Seebeck coefficient vs electrical conductivity plots of doped polymer films with and without QD. The solid lines are calculated curves based on the Kang–Snyder transport model (*s* = 3 is assumed). *σ*_E0_ value of each curve is also shown. The inset is an enlarged view of the squared part, and the *σ*_E0_ values of the solid lines in the inset are 0.1, 0.08, 0.06, and 0.04 S cm^−1^ in order from the top. The interior of data points obtained from the samples without QD is filled with yellow. b) UV–vis–NIR spectra of P3HT and P3HT‐QD films. Black and red: undoped samples. Navy and green: sequentially doped by AuCl_3_. c) ESR spectra of doped P3HT and doped P3HT‐QD films. d) AFM height images (top panels) and KPFM images (bottom panels) of P3HT and P3HT‐QD films, before and after doping with AuCl_3_.

The experimentally measured *S* and *σ* values of doped conjugated polymer films with different QD loadings are plotted in a |*S*| vs *σ* plot (**Figure** **5**a). Theoretically calculated *S*‐*σ* curves are also shown in the plot, assuming Kang–Snyder transport model which is represented by the following equation,:

(1) $$\sigma_{E}\;\left(E,T\right)=\left\{\begin{array}{c} \sigma_{E0}\left(T\right)\times {\left(\frac{E-E_{t}}{k_{B}T}\right)}^{s}\;\left(E>E_{t}\right)\\ 0\;(E<E_{t}) \end{array}\right.\;$$

where *σ_E_* is the transport function, *σ_E_*_0_ is a temperature‐dependent transport coefficient, *E*/*k*_B_ *T* is the reduced energy of the charge carriers, *E*_t_ is the transport level, and *s* is the transport parameter, which then results in the expressions for *σ* and *S* as:

(2) $$\sigma =\sigma_{E0}\;\times sF_{s-1}\left(\eta \right)$$

and:

(3) $$S=\frac{k_{B}}{e}\;\left[\frac{\left(s+1\right)F_{s}\left(\eta \right)}{sF_{s-1}\left(\eta \right)}-\eta \right]$$

respectively. Here, *F*_i_ is the complete Fermi‐Dirac integral, *e* is the elementary charge, and *η* is the reduced chemical potential, *η* = (*E*_F_−*E*_t_)/(*k*_B_ *T*).^[^ ^14^ ^]^ In the calculation of the *S*‐*σ* curves, we used *s* = 3, because it is known to well describe the transport property of most conjugated polymers including P3HT, PBTTT, and PDPP3T.^[^ ^14^ ^]^ According to the model, if the decomposed QD only changes the charge carrier concentration (i.e., doping level) and does not affect the transport model of the doped conjugated polymer film, the measured data points should exist on a single *S*‐*σ* curve with a specific *σ*_E0_. However, the measured data points do not align in this way; instead, their positions lie on a different curve with significantly different *σ*_E0_ values when decomposed QDs are present compared to when they are absent. This indicates that the decomposed QD significantly alters the charge transport properties of the doped polymer film, not limited to merely changing the doping level of the film.

The UV–vis–NIR spectra of the doped P3HT without QD and doped P3HT‐QD films showed similar spectral shapes and absorption intensities (Figure 5b), implying nearly the same polaron and bipolaron concentrations within them.^[^ ^47^ ^]^ Consistent results were also obtained from electron spin resonance (ESR) spectroscopy measurements, in which similar intensities were observed in the ESR spectra of the doped P3HT and doped P3HT‐QD films (Figure 5c). These results indicate that the charge carrier concentration and doping level of the doped P3HT were not significantly affected by the QD decomposition. Therefore, the QD‐induced change in the electrical conductivity and Seebeck coefficient can be attributed to changes in other factors that affect the charge transport in the doped polymer film, rather than a change in the doping level.

Various factors related to charge transport in the doped polymer film can be affected by decomposed QDs. For example, changes in the packing structure of P3HT, or ion‐induced generation of localized states^[^ ^48^, ^49^, ^50^ ^]^ can influence the density of states of doped P3HT, which may, in turn, alter its transport properties. While these factors do have important impacts on charge transport, they may not be sufficient to explain the significant enhancement of the Seebeck coefficient. Instead, we attribute the Seebeck coefficient enhancement to the energy filtering effect resulting from QD decomposition.

We speculated that the decomposition of QDs would result in the formation of local spatial regions where the ions from decomposed QDs are highly concentrated within the polymer film. Unlike the doped P3HT film without QDs containing only dopant anions (i.e., AuCl_4_^−^), the doped polymer‐QD film contained much greater amounts of additional cations and anions (e.g., Pb^2+^, Br^−^, Cl^−^, etc.) because of the decomposition of the CsPbBr_3_ QDs, particularly in the regions where the QDs were present before they decompose. The presence of such ion‐concentrated regions was expected to affect the electronic structure of the doped P3HT‐QD film. Indeed, when Kelvin probe force microscopy (KPFM) was performed on doped P3HT‐QD film to quantify its work function (WF) as a function of the position on the film surface, the presence of the ion‐concentrated regions and their WF deviating from that of the other parts of the film could be observed (Figure 5d). The undoped P3HT film has a WF of ≈4.08 eV which is uniform across the entire film. In the undoped P3HT‐QD film, the WF in the area where the CsPbBr_3_ QDs were present appeared to be slightly higher (≈4.14 eV) than that in the other regions without QDs (≈4.09 eV). After doping, both the P3HT and P3HT‐QD films showed significantly increased WF (≈5.6 eV) due to the p‐doping of P3HT. In the height image of doped P3HT film, spherical particles are present over the entire film surface, which are thought to be Au particles formed during the doping process by the reduction of the dopant. Despite the presence of these Au particles, the WF of doped P3HT film is nearly constant at ≈5.6 eV. In the height image of doped P3HT‐QD film, numerous sunken depressions are formed as the QD aggregates decompose and disappear during the sequential doping process. Notably, the WF map of the doped P3HT‐QD film shows significant nonuniformity owing to the presence of regions with lower work functions (0.06–0.13 eV lower) than the other regions; the locations of the low‐WF regions precisely coincide with those of the sunken depressions in the height image, strongly indicating that the relatively low WFs of these regions are the consequence of the decomposition of the QDs and the subsequent embedding of the resulting ions, although it is challenging to elucidate the composition and density of ions within these regions.

**Figure 6:**
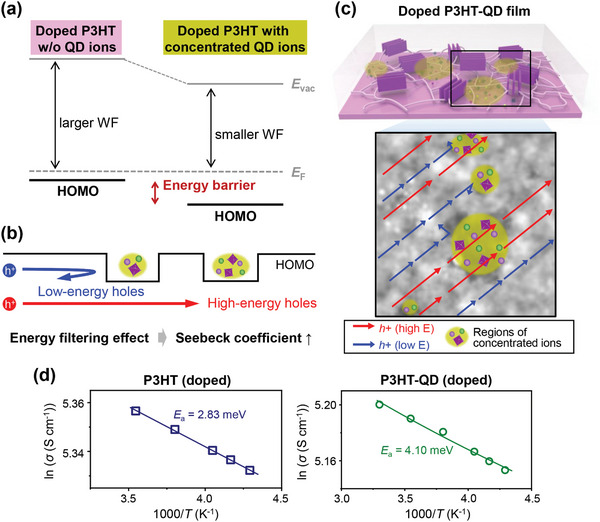
Suggested origin of QD‐induced enhancement of thermoelectric performance. a) Energy diagram of doped P3HT‐QD film drawn based on KPFM measurement results. An energy barrier for hole transport is formed between the regions with and without concentrated ions from QD. b,c) The energy filtering effect occurred by the energy barrier, which leads to the increase in the Seebeck coefficient. Low‐energy holes are filtered by the energy barrier and cannot be transported through the ion‐concentrated regions (blue arrows), while high‐energy holes can be transported in both regions with and without concentrated ions (red arrows). b) Illustration of the energy filtering effect using an energy diagram. c) Schematic illustration of the energy filtering effect expressed in the top view of the sample. d) Temperature dependence of electrical conductivity of doped P3HT films with and without QD. Data points represent experimentally measured values. Solid lines are the fitted curves using the equation *σ*(*T*) ∝ exp(−*E*_a_/*k*_B_ *T*).

The low WFs of the ion‐concentrated regions imply that the vacuum level in these regions is shifted to lower energies compared to other regions. The vacuum level shift then would lead to deeper HOMO energy levels in the ion‐concentrated regions than that in the other regions. As a result, an energy barrier is formed between them (**Figure** **6**a), affecting hole transport within the film. It has been widely reported that when an internal energy barrier is present within the charge transport pathway of thermoelectric material, the energy barrier impedes the transport of low‐energy carriers while allowing the transport of high‐energy carriers. This “energy filtering effect” therefore increases the mean energy of charge carriers contributing to the electrical conduction within the material, enhancing the Seebeck coefficient of the material.^[^ ^51^ ^]^ In this context, we expect that the energy barrier formed between the ion‐concentrated regions and the other regions will cause filtering of low energy holes (Figure 6b), which primarily contributes to the increased Seebeck coefficient of the doped P3HT‐QD film compared to that of doped P3HT film without QD. The mechanism of QD‐induced Seebeck coefficient enhancement is schematically depicted in Figure 6c.

The occurrence of the energy filtering effect in a conjugated polymer film is often characterized by the increased activation energy of charge carrier transport within the film.^[^ ^25^, ^26^, ^28^ ^]^ That is, the internal energy barrier that creates the energy filtering effect contributes to an increase in the overall activation energy for charge carrier transport within the film compared to the case in the absence of the energy filtering effect. Therefore, we investigated the temperature dependence of the electrical conductivity of the samples to compare the activation energy for charge transport (Figure 6d). The activation energy was quantified by fitting the data points with the Arrhenius‐type equation of the form *σ*(*T*) ∝ exp(−*E*_a_/*k*_B_ *T*), where *E*_a_ is the activation energy. Although the use of this equation does not reflect a detailed charge transport model or distinguish a specific charge transport mechanism, it can provide an estimation of the overall activation energy involved in the charge transport process, which is sufficient for our purpose. The estimated *E*_a_ values were 2.83 and 4.10 meV for doped P3HT and doped P3HT‐QD films, respectively. This result indicates a significant increase of activation energy for charge transport in the doped P3HT‐QD film due to the presence of the regions with concentrated ions from QDs, which is strong indirect evidence of the occurrence of the above‐mentioned energy filtering effect.

The decrease in the Seebeck coefficient at QD contents higher than the optimal content possibly originates from the excessive generation of ion‐concentrated regions and excessively high ion concentrations in these regions, which would severely increase the structural and energetic disorder of the entire doped polymer film.^[^ ^48^, ^50^ ^]^ In such cases, the electronic structure and charge transport mechanism would deviate far from those of doped polymer films without decomposed QDs or the films with optimal amounts of decomposed QDs.

Finally, we note that an explanation of the QD‐induced change in the Seebeck coefficient of doped IDTBT film (from a negative number to a more negative number) would require further investigations on thermoelectric properties of the film, including detailed discussions on polarity inversion of the Seebeck coefficient. Despite its p‐type transport characteristics in its pristine state, IDTBT showed an n‐type Seebeck coefficient in our experiments when it was doped and incorporated with QDs. Some recent studies have tried to unravel the underlying principles of polarity inversion of the Seebeck coefficient upon doping conjugated polymers, and have suggested explanations in terms of changes in the density of states and charge transport mechanisms of the polymers upon doping.^[^ ^52^, ^53^ ^]^ However, the polarity inversion in doped polymer films is still not fully understood, and therefore, we consider the more detailed discussion on our experimental results on IDTBT to be beyond the scope of the present study and would be dealt with in our future studies.

### 2.5 Thermoelectric Modules

**Figure 7:**
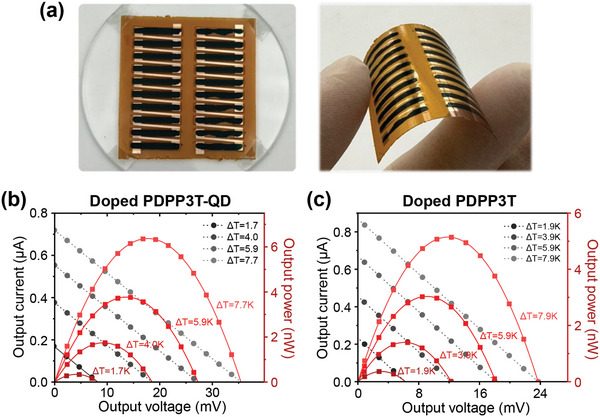
Thermoelectric module based on AuCl_3_ doped PDPP3T. a) Photos of the flexible thermoelectric module consisting of 10 legs. b) Output power curves of the module based on doped PDPP3T‐QD at various temperature differences. c) Output power curves of the module based on doped PDPP3T without QD at various temperature differences.

Flexible organic thermoelectric modules were fabricated using doped PDPP3T with and without CsPbBr_3_ QDs (**Figure** **7**). The photos of the fabricated modules are shown in Figure 7a. It consists of ten legs connected in series and has lateral ∏‐type structure. The detailed module fabrication process is described in Supporting Information. The output powers of the modules were measured in the lateral direction at various temperature gradients. At a temperature difference of ≈8 K, the module based on doped PDPP3T‐QD film showed an open‐circuit voltage (*V*_OC_) of 36 mV and short‐circuit current (*J*_SC_) of 0.72 µA, exhibiting maximum power output of 6.4 nW (Figure 7b). The active area of the module was 1.05 cm^−2^ (0.105 cm^2^ × 10), so the maximum power density of the module was 6.1 nW cm^−2^. The module based on doped PDPP3T without the QDs showed the *V*_OC_ and *J*_SC_ of 24 mV and 0.87 µA, respectively, exhibiting maximum output power and power density of 5.2 nW and 5.0 nW cm^−2^ (Figure 7c). The higher *V*_OC_ and smaller *J*_SC_ of the module with doped PDPP3T‐QD compared to those of doped PDPP3T are consistent with the results shown in Section 2.2, where the Seebeck coefficient was higher and the electrical conductivity was lower in the doped PDPP3T‐QD film than in the doped PDPP3T film. Note that the modules were fabricated by drop‐casting the PDPP3T or PDPP3T‐QD solutions; thus, the film thickness and microstructure deviated from the optimal conditions. Nevertheless, the modules clearly showed a difference in their thermoelectric performance, i.e., ≈23% enhancement in the maximum output power by introducing CsPbBr_3_ QDs. These results confirm the effectiveness of the proposed method for the development of high‐performance organic thermoelectric modules.

We further fabricated a thermoelectric module with a rolled‐up structure (Figure S5, Supporting Information). In general, rolled‐up structured thermoelectric modules can have a greater areal number density of thermoelectric legs compared to conventional ∏‐structured modules and therefore is beneficial in achieving a greater output power density.^[^ ^54^ ^]^ Our prototypical rolled‐up‐structured module was fabricated following nearly the same procedure as the lateral ∏‐structured module, except that it consisted of four thermoelectric legs and was fabricated on a substrate with a higher aspect ratio. The module was then rolled up to be completed (Figure S5a, Supporting Information). For measurements, the module was laid down so that the upper and lower sides of the module were in contact with the vertically arranged hot and cold Peltier modules, respectively (Figure S5b, Supporting Information). The module exhibited a maximum power output of 24 pW when a temperature difference of 6 K was applied. Considering the effective area of the module (1.8 cm^2^), estimated as the product of the length of the cylinder and the diameter of the base plane, this corresponded to a power density of 13.4 pW cm^−2^. Further optimization of the module structure is expected to improve the thermoelectric performance of the module.

## 3 Conclusion

In this study, we demonstrated a novel and effective strategy for enhancing the thermoelectric performance of conjugated polymers. By incorporating inorganic perovskite QDs into a conjugated polymer film and subsequent decomposition via sequential solution doping, we successfully embedded additional ions into the doped polymer thin film. The ions from the QDs were concentrated near the original QD locations during the decomposition process, forming regions where they were concentrated. At the optimal QD content, the presence of ion‐concentrated regions did not affect the doping level of the polymer film but caused a mild decrease in electrical conductivity and a significant increase in the Seebeck coefficient of the doped polymer films. Because of the QD‐induced increase in the Seebeck coefficient, the doped polymer‐QD film exhibited a significantly higher thermoelectric PF than the doped polymer film without QDs. The boosted Seebeck coefficient was attributed to the energy filtering effect caused by the energy barrier formed between the ion‐concentrated regions and the other regions in doped polymer‐QD film. It was also shown that this strategy could be applied universally across various conjugated polymers, whereas the degree of enhancement of the Seebeck coefficient and the PF enhancement varied depending on the polymer.

The strategy presented in this study opens a new opportunity to control the thermoelectric properties of conjugated polymers by incorporating ionic compounds into them. This has rarely been attempted because of processing difficulties, especially because of the low solubility mismatch and poor compatibility between the polymers and ionic compounds in a solvent. As demonstrated in this study, the passivation of halide perovskite QDs with ligands ensures their processability in organic solvents and effective dispersion in a conjugated polymer. Furthermore, by utilizing the chemistry of the AuCl_3_ dopant, polymer doping, and QD decomposition were simultaneously achieved, resulting in the fabrication of doped conjugated polymers incorporating additional ions.

We expect that the materials used in this strategy can be expanded beyond perovskite QDs and AuCl_3_ to include other ionic compounds and dopants capable of producing similar effects. For example, it has been reported that various metal and inorganic nanocrystals can be dissociated in the presence of halide ions, as these ions react with the surface atoms of the nanocrystals to form more stable complexes.^[^ ^55^ ^]^ Therefore, provided that the nanocrystal can be well incorporated into conjugated polymer films and can be well dissociated during the sequential doping process with dopants containing halide ions, the nanocrystal‐dopant pair could also be a good candidate applicable to the strategy presented in this work. Exploring which ionic compounds and dopant pairs, other than CsPbBr_3_ QDs and AuCl_3_, could lead to a more effective increase in the Seebeck coefficient of conjugated polymers would be a valuable topic for future research.

## Conflict of Interest

The authors declare no conflict of interest.

## Supporting information



Supporting Information

## Data Availability

The data that support the findings of this study are available from the corresponding author upon reasonable request.
