# Supplementary material for: “Popping the Ion‐Basket”: Enhancing Thermoelectric Performance of Conjugated Polymers by Blending with Latently Dissociable Perovskite Quantum Dots
Source: Adv Sci (Weinh). 2025 Jan 22;12(11):2412663. doi: 10.1002/advs.202412663 (PMC11924034; doi:10.1002/advs.202412663)
Supplement: Supplementary file 1 — Supporting Information [file ADVS-12-2412663-s001.docx]

Supporting Information

**“Popping the Ion-Basket”: Enhancing Thermoelectric Performance of Conjugated Polymers by Blending with Latently Dissociable Perovskite Quantum Dots**

*Hansol Lee,^†^ Hoimin Kim,^†^ Haedam Jin, Seungju Kang, Tae Woong Yoon, Dongki Lee, Guobing Zhang, Min Kim,* and Boseok Kang**

Prof. H. Lee

Department of Chemical and Biological Engineering, Gachon University, Seongnam 13120, Republic of Korea

H. Kim, S. Kang, T. W. Yoon, Prof. B. Kang

SKKU Advanced Institute of Nanotechnology (SAINT) and Department of Nano Science and Technology, Sungkyunkwan University, Suwon 16419, Republic of Korea

Email: bskang88@skku.edu

Haedam Jin

Graduate School of Integrated Energy-AI, Jeonbuk National University, Jeonju 54896, Republic of Korea

Prof. B. Kang

Department of Nano Engineering and Department of Semiconductor Convergence Engineering, Sungkyunkwan University, Suwon 16419, Republic of Korea

Email: bskang88@skku.edu

Prof. M. Kim

School of Chemical Engineering and School of Semiconductor and Chemical Engineering, Jeonbuk National University, Jeonju 54896, Republic of Korea

Email: minkim@jbnu.ac.kr

Prof. D. Lee

Department of Nanotechnology and Advanced Materials Engineering, Sejong University, 209 Neungdong-ro, Gwangjin-gu, Seoul 05006, Republic of Korea

Prof. G. Zhang

National Engineering Lab of Special Technology, Academy of Optoelectronic Technology, Anhui Province Key Laboratory of Measuring Theory and Precision Instrument, School of Chemistry and Chemical Engineering, Key Laboratory of Advance Functional Materials and Devices of Anhui Province, Hefei University of Technology, Hefei 230009, China

^†^These authors contributed equally to this work.

*Corresponding authors.

**Table of Contents**

**1. Experimental Section**

**2. Supplementary Figures**

**Figure S1.** EDS elemental maps of P3HT-QD film

**Figure S2.** UV-vis absorption and PL spectra of neat CsPbBr_3_ QD films

**Figure S3.** SEM images of neat CsPbBr_3_ QD films

**Figure S4.** GIXD measurements on neat CsPbBr_3_ QD films

**Figure S5.** Rolled-up structured thermoelectric module

**3. Supplementary Tables**

**Table S1.** Thermoelectric properties of AuCl_3_-doped P3HT-QD films annealed at different temperatures.

**Table S2.** Effect of adding OA ligand on thermoelectric properties of P3HT film.

**References for Supporting Information**

**1. Experimental Section**

**1.1. Materials**

Poly(3-hexylthiophene) (P3HT) was purchased from Tokyo Chemical Industry (TCI) Co., Ltd. 2,1,3-benzothiadiazole-4,7-diyl-co-4,4,9,9-tetrahexadecyl-4,9-dihydro-s-indaceno[1,2-b:5,6-b‘]dithiophene-2,7-diyl (IDTBT) was obtained from Derthon Optoelectronics Materials Science Technology Co., Ltd. Poly[2,5-bis(3-tetradecylthiophen-2-yl)thieno[3,2-b]thiophene] (PBTTT), gold(III) chloride (AuCl_3_), and hexamethyldisilazane (HMDS) were purchased from Sigma-Aldrich. Poly{2,2′-[(2,5-bis(2-octyldodecyl)-3,6-dioxo-2,3,5,6- tetrahydropyrrolo[3,4-c]pyrrole-1,4-diyl)dithiophene]-5,5′-diyl-alt-thiophen-2,5-diyl} (PDPP3T) and CsPbBr_3_ were synthesized as described in the sections below. The solvents used to dissolve the polymers, dopants, and cleaning substrates were purchased from Sigma-Aldrich. For the synthesis of CsPbBr3 quantum dots (QDs), cesium carbonate (Cs_2_CO_3_, 99.995 %), 1-octadecene (ODE, 90 %), oleic acid (OA, technical grade, 90%), oleylamine (OLA, technical grade, 70%), ethyl acetate (anhydrous, 99.8 %), and hexane (anhydrous, 95 %) were purchased from Sigma-Aldrich. Lead bromide (PbBr_2_, 98 %) was purchased from TCI.

**1.2. Synthesis of PDPP3T**

The polymer was synthesized by Pd-catalyzed Stille coupling under microwave irradiation. To a 30 mL microwave vial was added 3,6-bis(5-bromothiophen-2-yl)-2,5-bis(2-octyldodecyl)pyrrolo[3,4-c]pyrrole-1,4(2H,5H)-dione (0.244 g, 0.24 mmol), 2,5-bis(trimethylstannyl)thiophene (0.99 g, 0.24 mmol), tris(dibenzylideneacetone)dipalladium (4.4 mg), Tri(o-tolyl)phosphine(5.85 mg) and anhydrous chlorobenzene (6 mL). The solution was heated to 200 °C and reacted for 10 min, followed by cooling to 70 °C. To cap the unreacted end groups of the polymer, the reaction vial was additionally charged by 2-bromothiophene (0.5 mL), heated to 200 °C, reacted for 3 min, and cooled to 70 °C, followed by the same sequence with 2-(tributylstannyl)thiophene (0.38 ml). The resulting polymers were precipitated using methanol (300 mL). The resulting solid was filtered, washed with methanol, and dried. The solid was further purified by Soxhlet extraction using acetone, methanol, dichloromethane, and n-hexane, and then dissolved in chloroform. After the extraction with CHCl_3_ the polymer was collected and dried under reduced pressure. A dark green solid of PDPP3T was obtained after removing the solvent (0.140 g, 58.3 %).

**1.3. Synthesis of perovskite QDs**

CsPbBr_3_ perovskite QD were synthesized according to a previously reported method.^[1-3]^ Cesium oleate was prepared by transferring Cs_2_CO_3_ (1.2 mmol), OA (1.2 mL) and ODE (15 mL) into a three-neck flask and degassed under vacuum for 10 min at 120 °C. The flask was then filled with N_2_ gas for 10 min. The solution was then gassed/degassed thrice. Subsequently, the temperature was increased to 150 °C until a clear solution was obtained. The obtained Cs-oleate was stored in a glove box and heated to 150 °C before use. Lead halide precursor was prepared by adding PbBr_2_ (1.08 mmol), ODE (30 mL), OLA (3.3 mL) and OA (6 mL) into a three-neck flask and degassed under vacuum for 10 min at 120 °C, and the flask was then filled with N_2_ for 10 min. The solution was then gassed/degassed thrice. Then, the temperature was increased to 170 °C, and 1.8 mL of the heated Cs-oleate solution at 150 °C was quickly injected. After 5 s, the solution was cooled in an ice-water bath. The crude solution was centrifuged at 8000 rpm for 5 min. The precipitate was then redispersed in hexane (10 mL). The solution was then centrifuged at 5000 rpm for 5 min. The precipitate was discarded, and the supernatant was collected to obtain perovskite particles. Subsequently, ethyl acetate (20 mL) was added and the solution was centrifuged at 9000 rpm for 5 min. Finally, the precipitate was re-dispersed in hexane.

**1.4. Preparation of the pristine polymer and polymer-QD blend films and their doping process**

The substrates (glass and silicon wafers) were cleaned by ultrasonication with acetone, isopropyl alcohol, and deionized water. After cleaning, the substrates were dried and treated with UV-ozone for 20 min. Polymer and perovskite QD were blended at a fixed ratio, dissolved in anhydrous chlorobenzene at 7 mg ml^-1^, and stirred for 12 hours at 150°C. The resulting solution was then spin-coated onto the substrate (2000 rpm, 60s) and annealed at 150°C for 10 min before slowly cooled down. For the doped films, an AuCl_3_ solution (acetonitrile, 1 mg ml^-1^) was dropped onto the blended film, and after 1 min, it was spin-coated at 5000 rpm for 60 s. To completely dry the remaining solvent, the films were baked at 80 °C for 10 minutes. Except for substrate cleaning, all processes were performed in an N_2_-filled glovebox.

**1.5. Electrical and thermoelectrical characterizations**

Electrical conductivity was determined using the van der Pauw method with a Keithley 4200 instrument. The Seebeck coefficients were measured using a custom-made setup. This experimental arrangement involved two Peltier modules that generated a temperature gradient, along with a thermocouple that concurrently recorded both the temperature variation and the resulting thermovoltage. To achieve this, the currents in the Peltier modules were controlled using two Keithley 2460 sources. The entire process was facilitated by integrating a Keithley 6510/7700 Data Acquisition System and the Keithley 2182A nanovoltmeter.

**1.6. Other characterizations**

Ultraviolet-visible-near-infrared (UV-vis-NIR) absorption spectra were acquired using a V-770 spectrometer (JASCO) and analyzed in the wavelength range of 330–2500 nm at intervals of 1 nm. Photoluminescence spectroscopy was performed using FluoroMax Plus (HORIBA Scientific), measuring the wavelength range from 400 to 600 nm with 365 nm excitation. Scanning electron microscopy (SEM) and energy-dispersive X-ray spectrometry (EDS) images were obtained using a JSM-IT800 instrument (JEOL). Electron Spin Resonance (ESR) spectra were acquired using a JES-X320 instrument (JEOL). Atomic force microscopy (AFM) and Kelvin-probe force microscopy (KPFM) were performed using an NX-10 instrument (Park Systems). The work function of the AFM tip (NCS36/Cr-Au probe, Park Systems) was calibrated before and after every measurement with highly ordered pyrolytic graphite, whose work function is well known to be 4.6 eV. The work function of each sample was calculated from the measured surface potential of each sample and the tip work function. Transmission electron microscopy (TEM) samples were analyzed using a JEM-ARM200F instrument (JEOL) at the Center for University-wide Research Facilities (CURF) of Jeonbuk National University. X-ray diffraction (XRD) patterns were obtained using MAX-2500 (Rigaku) with a Cu K_α_ source at the Future Energy Convergence Core Center (FECC) of Jeonbuk National University. GIXD measurements were conducted at the 3C and 9A beamlines of the Pohang Accelerator Laboratory (PAL).

**1.7. Thermoelectric module fabrication**

To generate the module pattern, an Image Master 350PC Smart dispenser (MUSASHI) was employed with a 32 G needle featuring a 100 μm inner diameter. For the deposition of contact electrodes and the n-type thermoelectric leg, Ti and Au were sequentially thermal-evaporated (4 nm and 25 nm, respectively) onto a polyimide film affixed to glass substrate at rates of 0.2 and 1 Å s^−1^, respectively. To achieve a uniform coating of subsequent layers on the film, the polyimide film was treated with HMDS (Sigma-Aldrich) to form a self-assembled monolayer. The HMDS solution was spin-coated (3000 rpm, 30 s) onto a polyimide film and baked at 150 °C in a vacuum oven for 1 h. The polyimide film was washed with ethanol and dried. For the precise pattern printing of the polymer (or polymer-QD blend) solution in the designated area, Teflon ink was predispensed as a bank layer. A 1 wt% Teflon solution (AF 1600, Dupont), dissolved in a fluorinated solvent (Fluorinert^TM^ FC-40, Sigma-Aldrich), was dispensed to the bank layer at a printing speed of 5 mm s^-1^ and a dispensing pressure of 0.1 kPa).^[4]^ The bank layer was dried at 100 °C for 30 min. Then, a polymer (or polymer-QD blend) solution was printed on the film at the printing speed of 5 to 10 mm s^-1^ and dispensing pressure of 10 kPa, and was dried at 100 °C for 10 minutes. For doping, AuCl_3_ solution (acetonitrile, 1 mg ml^-1^) was dropped onto the polymer (or polymer-QD) film. After 1 min, the AuCl_3_ solution was spin coated at 5000 rpm for 60 s. Finally, the films were baked at 80 °C for 10 min.

**2. Supplementary Figures**

**
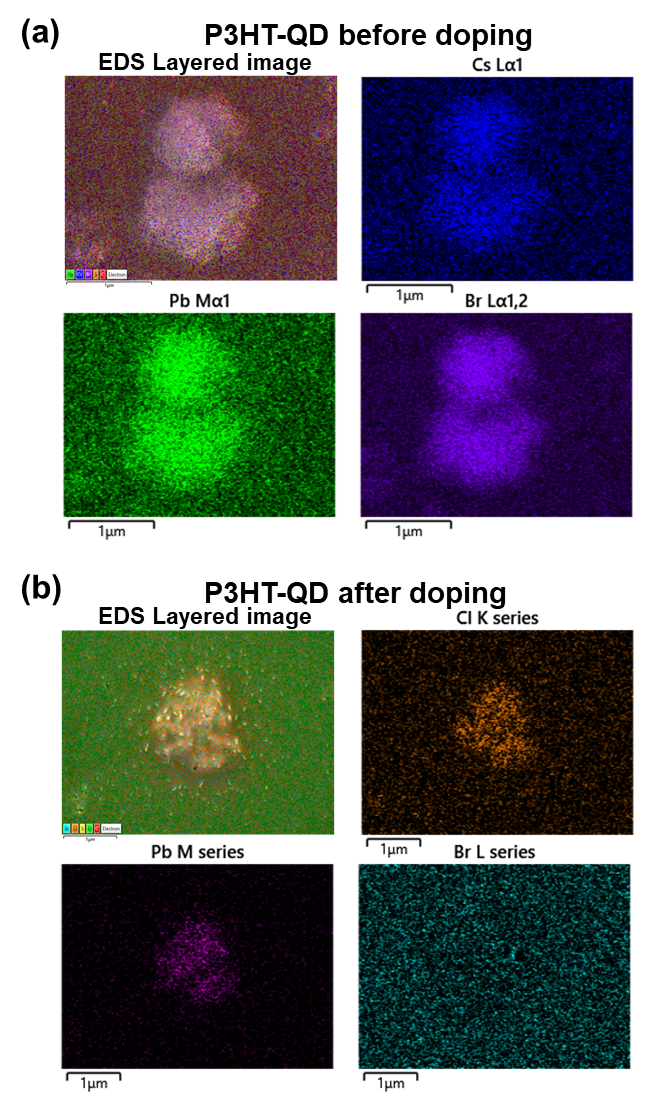
**

**Figure S1.** Elemental maps of P3HT-QD blend films (a) before and (b) after sequential doping with AuCl_3_ solution. In (b), the doping condition was adjusted to capture the image of the QD undergoing decomposition (i.e., not completely decomposed).

**
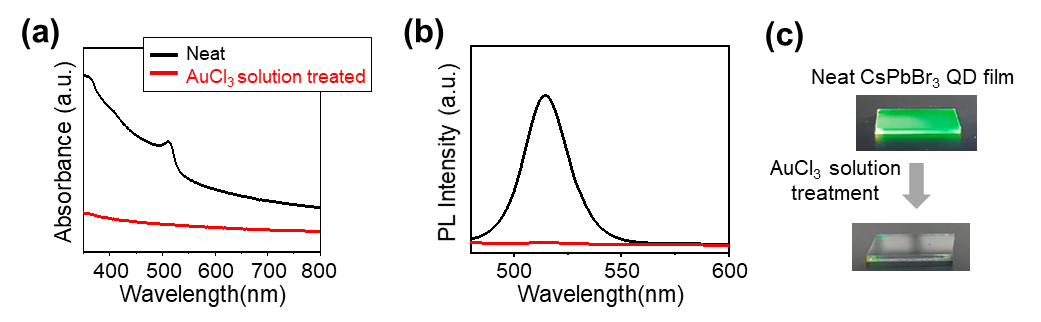
**

**Figure S2.** (a) UV-vis absorption spectra of neat CsPbBr_3_ QD films before (black) and after (red) the treatment with AuCl_3_ dopant solution. (b) PL spectra (excited at 365 nm) of neat CsPbBr_3_ QD films before (black) and after (red) the treatment with AuCl_3_ dopant solution. (c) Photos of the neat CsPbBr_3_ QD films emitting green light. After treatment with AuCl_3_ solution, the film does not emit light any more due to decomposition of the CsPbBr_3_ QDs.

**
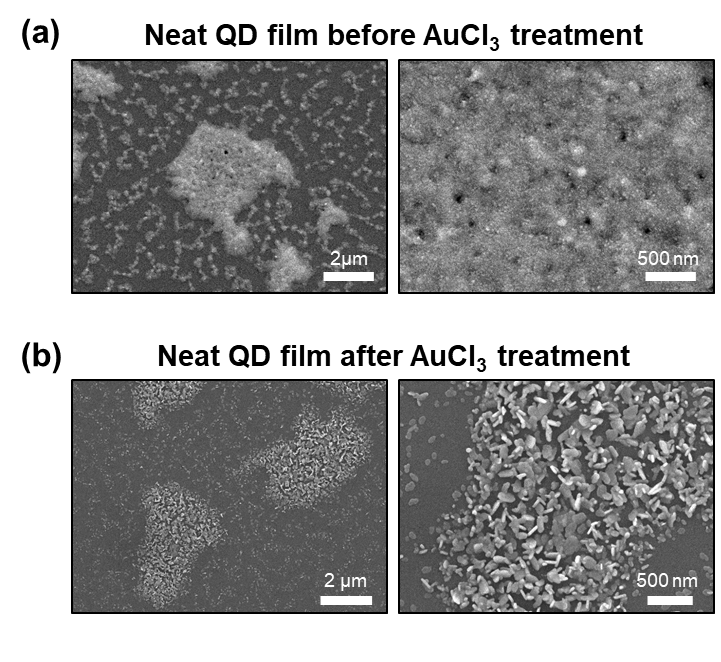
**

**Figure S3.** SEM images of neat CsPbBr_3_ QD films (a) before and (b) after the treatment with AuCl_3_ dopant solution.

**
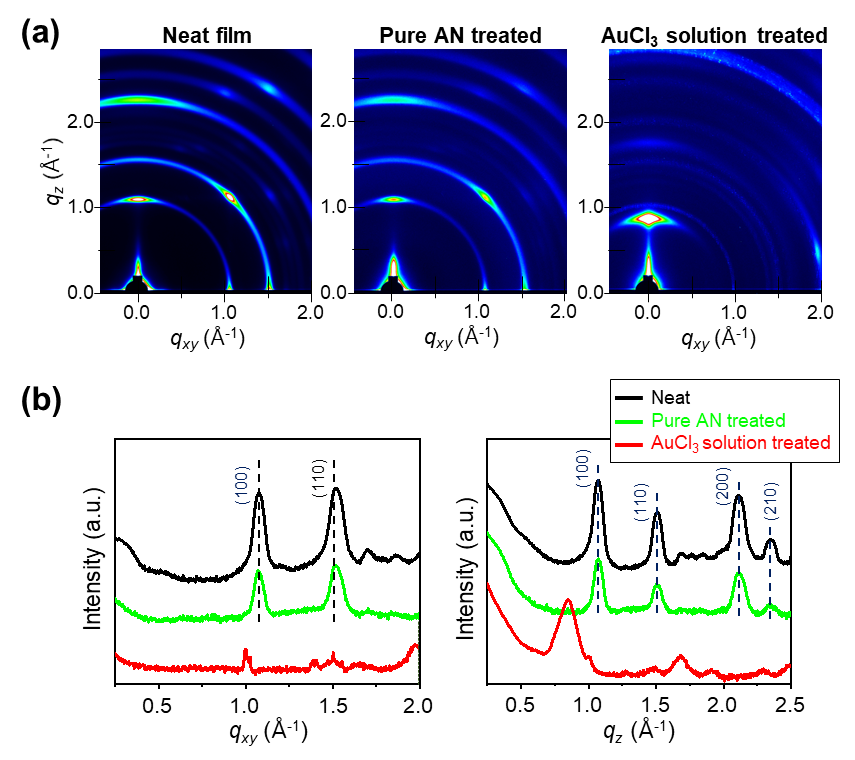
**

**Figure S4.** GIXD measurements on neat CsPbBr_3_ QD films before and after the treatment with pure AN or AuCl_3_ solution. (a) 2D images of the diffraction pattern. (b) The corresponding 1D line profiles along the in-plane (left) and out-of-plane (right) directions.

**
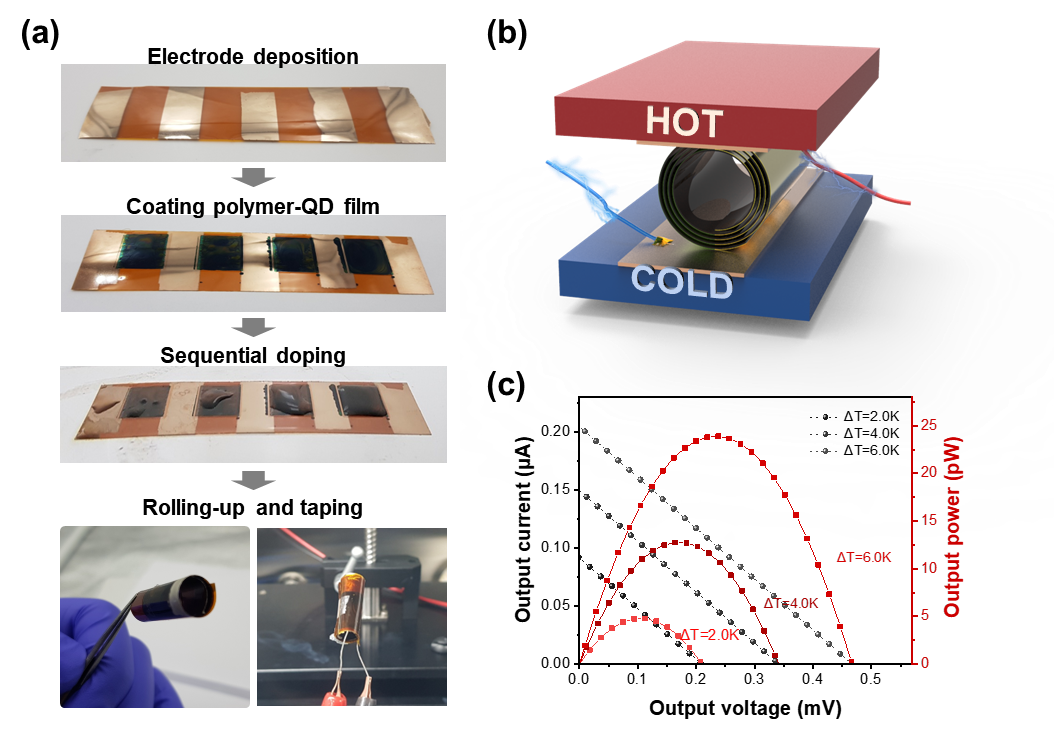
**

**Figure S5.** Rolled-up structured thermoelectric module based on AuCl_3_-doped PDPP3T. (a) Fabrication steps and actual photos of the fabricated module. The module consists of 4 legs. (b) Schematic of the module structure and its measurement. (c) Output power curves of the module at various temperature differences. The maximum output power is ~24 pW (at Δ*T* = 6.0 K).

**3. Supplementary Tables**

**Table S1.** Thermoelectric properties of AuCl_3_-doped P3HT-QD films thermally annealed at different temperatures before doping.

| Polymer | QD content  [wt%] | Annealing temperature  [°C] | Electrical  Conductivity  [S cm^-1^] | Seebeck  coefficient  [μV K^-1^] | Power factor  [μW m^-1^ K^-2^] |
| --- | --- | --- | --- | --- | --- |
| P3HT | 0 | 150 | 246.0 ± 10.2 | 52.5 ± 0.3 | 73.9 |
|  |  | 180℃ | 261.5 ± 3.3 | 54.5 ± 1.0 | 72.1 |
|  | 20 | Not annealed | 243.9 ± 10.0 | 59.4 ± 1.2 | 86.1 |
|  |  | 150℃ | 253.4 ± 2.5 | 59.2 ± 1.6 | 88.8 |
|  |  | 180℃ | 272.0 ± 2.1 | 56.5 ± 1.5 | 86.8 |
|  |  | 210℃ | 246.1 ± 5.2 | 57.0 ± 1.3 | 80.0 |
|  | 30 | Not annealed | 222.3 ± 6.5 | 63.0 ± 1.0 | 88.2 |
|  |  | 150℃ | 236.9 ± 1.4 | 59.5 ± 0.3 | 83.9 |
|  |  | 180℃ | 237.1 ± 3.1 | 59.0 ± 3.3 | 82.5 |
|  |  | 210℃ | 234.5 ± 4.4 | 57.1 ± 1.2 | 76.2 |
|  | 40 | Not annealed | 169.9 ± 7.0 | 63.9 ± 1.5 | 69.4 |
|  |  | 150℃ | 222.8 ± 6.3 | 57.9 ± 0.2 | 74.7 |
|  |  | 180℃ | 195.5 ± 2.1 | 58.5 ± 2.5 | 66.9 |
|  |  | 210℃ | 224.1 ± 3.1 | 57.7 ± 0.5 | 74.6 |
|  | 50 | Not annealed | 179.9 ± 7.0 | 56.1 ± 0.5 | 56.5 |
|  |  | 150℃ | 198.2 ± 2.6 | 59.0 ± 0.2 | 69.0 |
|  |  | 180℃ | 201.0 ± 15.2 | 55.7 ± 2.3 | 62.4 |
|  |  | 210℃ | 200.8 ± 2.0 | 55.7 ± 0.8 | 62.3 |

**Table S2.** Effect of adding OA ligand on thermoelectric properties of P3HT film.

| Polymer | Dopant | OA addition | Electrical  Conductivity  [S cm^-1^] | Seebeck  coefficient  [μV K^-1^] | Power factor  [μW m^-1^ K^-2^] |
| --- | --- | --- | --- | --- | --- |
| P3HT | AuCl_3_ | No | 246.0 | 52.5 | 73.9 |
|  | None | Yes | 0.002 | - | - |
|  | AuCl_3_ | Yes | 153.7 | 50.1 | 38.6 |

**References for Supporting Information**

[1] H. Jin et al. Eco-friendly Solvent-Processible and highly luminescent perovskite nanocrystals with polymer zwitterions for Air-Stable optoelectronics. *Chem. Eng. J.* **2023**, *459*, 141531.

[2] L. Protesescu et al. Nanocrystals of cesium lead halide perovskites (CsPbX_3_, X= Cl, Br, and I): novel optoelectronic materials showing bright emission with wide color gamut. *Nano lett.* **2015**, *15*, 3692.

[3] C. Lu et al. Cesium oleate precursor preparation for lead halide perovskite nanocrystal synthesis: The influence of excess oleic acid on achieving solubility, conversion, and reproducibility. *Chem. Mater.* **2019**, *31*, 62.

[4] Y.-S. Kim et al. Latent and controllable doping of stimuli-activated molecular dopants for flexible and printable organic thermoelectric generators. *Chem. Eng. J.* **2023**, 470, 144129.
